# Evaluation of the genetic relatedness of *Bacteroides fragilis* isolates by TRs analysis

**DOI:** 10.22038/ijbms.2020.35816.8532

**Published:** 2020-10

**Authors:** Niloofar Khodaei, Behrooz Sadeghi Kalani, Maryam Zamani, Rokhsareh Mohammadzadeh, Malihe Talebi, Tahmine Narimani, Negar Narimisa, Faramarz Masjedian Jazi

**Affiliations:** 1 Microbial Biotechnology Research Center, Iran University of Medical Science, Tehran, Iran; 2 Department of Microbiology, School of Medicine, Iran University of Medical Sciences, Tehran, Iran; 3 Clinical Microbiology Research Center, Ilam University of Medical Sciences, Ilam, Iran; 4 Department of Medical Microbiology, Faculty of Medicine, Ilam University of Medical Sciences, Ilam, Iran; 5 Department of Microbiology, School of Medicine, Isfahan University of Medical Sciences, Tehran, Iran

**Keywords:** Bacteroides fragilis, MLVA, PCR amplification, Tandem repeats, Typing

## Abstract

**Objective(s):**

Human gastrointestinal tract harbors a variety of bacteria with vital roles in human health. *Bacteroides fragilis* is considered one of the dominant constituents of gut microflora which can act as an opportunistic pathogen leading to various diseases, including colon cancer, diarrhea, uterine and intrathecal abscesses, septicemia, and pelvic inflammation. In this study, multiple locus variable number of tandem repeats analysis (MLVA) was performed to genetically differentiate 50 *B. fragilis* isolates.

**Materials and Methods:**

Eight suitable tandem repeats (TRs) were selected by bioinformatics tools and were then subjected to PCR amplification using specific primers. Finally, MLVA profiles were clustered using BioNumerics 7.6 software package.

**Results:**

All VNTR loci were detected in all isolates using the PCR method. Overall, *B. fragilis* isolates were differentiated into 27 distinct MLVA types. The highest diversity index was allocated to TR1, TR2, TR5, TR6, and TR8; with this taken into account, strain type 14 was the most prevalent with 12 strains belonging to this type. Clustering revealed three major clusters of A, B, and C. With regards to the pathogenicity of *B. fragilis* and the outcomes of infections related to this microorganism, it is imperative to study this microorganism isolated from both patients and healthy individuals.

**Conclusion:**

This study aimed at evaluating the efficiency of MLVA for the genetic differentiation of *B. fragilis*. The results of this study indicate the promising efficiency of MLVA typing for cluster detection of this bacterium.

## Introduction

*Bacteroides fragilis* is a Gram-negative, anaerobic bacillus residing in the human gut microflora (1). This anaerobic microorganism is regularly isolated from human infections causing serious complications due to the lack of proper antimicrobial therapy. Two molecular subtypes have been attributed to this microorganism: non-toxigenic *B. fragilis *(NTBF) and enterotoxigenic *B. fragilis *(ETBF). The latter subtype is considered an intestinal microorganism contributing to inflammatory bowel disease and colorectal cancer. Reportedly, antimicrobial resistance in this bacterium is increasing worldwide (2-4). 

Gut microflora can play vital roles in human health. The overgrowth of anaerobic gut microbiota, especially Bacteroides, can lead to several health outcomes including colon cancer, diarrhea, intrathecal and uterine abscesses, and pelvic inflammation. About 99% of bacterial gut microflora are anaerobic, 20-30% of which are in the Bacteroides group. Moreover, recent reports indicate increased antibiotic resistance levels to methicillin, cephalosporins, tetracycline, and clindamycin (5-7). 

Bacteria are often in a commensal relationship with humans; however, pathogenic bacteria can be observed infrequently. Colonization of commensal bacteria is advantageous for human health as it can lead to mucosal and systemic immunity (8, 9).

Gut microbiota can induce maturity of the host immunity and subsequently provide protection against various infections. In spite of the fact that *B. fragilis* constitutes less than 1% of intestinal flora, studies on animal models with colitis have demonstrated that owing to the possession of polysaccharide capsules, this microorganism can rectify the inadequacy of immune system caused by the absence of bacterial colonization (10, 11).

There are two types of polysaccharide capsules, including polysaccharide A (PSA) and polysaccharide B (PSB), both belonging to zwitterion polysaccharides (ZPSs) with both positive and negative charges on each residue. These capsular polysaccharides have been shown to induce TCD4+ immune responses, hence restricting the colonization of other pathogens and the spread of infections (12-14).

In certain conditions, however, *B. fragilis* can cause inflammatory bowel disease, intestinal abscesses, peritonitis, genital infections, deep ulcers, bone marrow infections, pediatric cellulitis and pneumonia, colon cancer, bacteremia, brain abscess, meningitis, and septic arthritis. Recent advances in molecular typing and investigating genomic polymorphism have significantly improved our understanding of bacterial evolution, pathogenicity, and reproduction. Today, Pulsed Field Gel Electrophoresis (PFGE) is considered the gold standard method for the epidemiological studies of *B. fragilis* in a short period. In this technique, banding patterns of bacterial whole genome are compared after being restricted by a unique-cutting DNA enzyme (15).

 Considering high costs and difficulty in analysis, PFGE should be replaced by other typing techniques (16). Another typing technique is Multi Locus Sequence Typing (MLST), which uses the sequencing of seven housekeeping genes and is both expensive and time-consuming (17). Another frequently used typing method is multiple locus variable number of tandem repeats analysis (MLVA) typing which is according to variable copy numbers of tandem repeats (VNTR). This method has been employed to discriminate bacterial species, including *Escherichia coli* (18, 19), Listeria (20), Brucella (20), and Staphylococci, and to determine any genetic relatedness among the isolates. 

This PCR-based technique identifies the number of replicates in a specific locus of bacterial genomes. After selecting the desired loci and designing specific primers, PCR is performed for the extracted bacterial DNA. Then, the size of PCR products is determined by electrophoresis and the number of repeats is counted by sequencing (21).

Due to the difficulties and limitations of studying anaerobic bacteria in Iran, few studies have investigated the genetic characteristics and typing of these bacteria. This study aimed at investigating MLVA for *B. fragilis* as a novel technique for typing and genetic differentiation of this microorganism. For this aim, suitable tandem repeats were selected to determine genetic diversity and conduct phylogenetic analysis. 

## Materials and Methods

***Bacterial collection and identification***

In total, 50 non-toxigenic *B. fragilis* isolates were attained from the previous study (22) and were stored in the microbial bank in the Department of Microbiology, Iran University of Medical Sciences, Tehran, Iran.

***Identification of VNTR loci***

The sequence of the genome of *B. fragilis YCH46* was obtained using GeneBank NCBI and tandem repeat sequences were analyzed using Tandem Repeats Finder (TRF version 4.09) which have been illustrated in Table 1 (23).

***Primer design***

Suitable specific primers were designed using Oligo 7, AlleleID, Primer3, and Oligo Analyser software packages. Table 2 shows the oligonucleotide primers and flanking regions of every VNTR locus. Annealing temperatures of the primers were determined *in silico *using the Primer-BLAST tool in the NCBI website. 

***Data analysis***

MLVA profiles were clustered using the BioNumerics 7.6 software package, with UPGMA (Unweighted Pair Group Method with Arithmetic mean) method. The similarity coefficient of Pearson’s correlation besides the minimum spanning tree (MST) was determined using the BioNumerics software package. The polymorphism index of the individual or combined VNTR loci was measured using the Hunter-Gaston diversity index (24).

## Results

**Figure 1:**
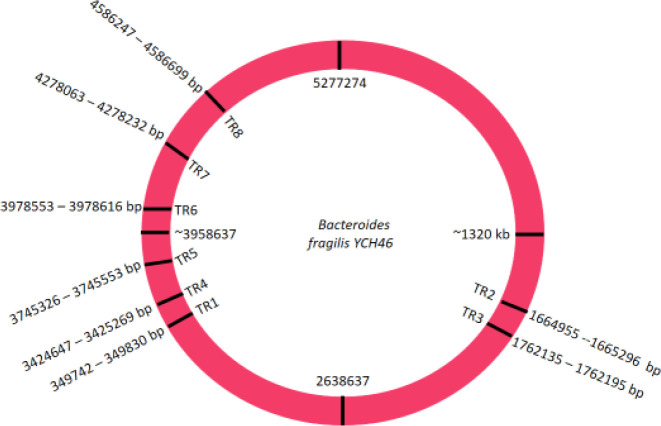
Genomic locations of tandem repeats (TR) of *Bacteroides fragilis* YCH46 in this study

MLVA typing was performed on 50 *B. fragilis* isolates originating from human stool. Eight regions were chosen for analysis based on the location of the genome, repeat length, percent of matches, and percent of indels between adjacent copies and copy numbers as indicated in Table 1 and Figure 1. The size of the repeat unit of the five VNTRs was in the range of 12 bp and 80 bp.

TR1, TR3, and TR5 are located in a non-coding region of the bacterial genome. TR2, TR4, TR6, and TR7 are located in a genomic region with a hypothetical protein function. Finally, TR8 is located in a region of the bacterial genome which is a ribosomal large subunit acting as pseudouridine synthase B. 

**Figure 2:**
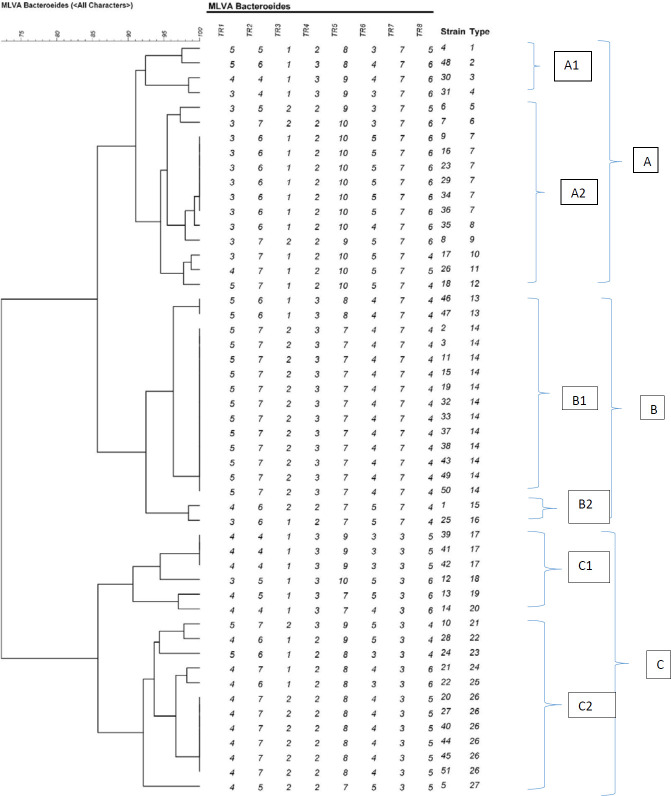
Multiple locus variable number of tandem repeats analysis (MLVA) clustering of the 50 *Bacteroides fragilis* isolates generated by the UPGMA algorithm. The names of isolates, the origin of isolates, MLVA profiling is shown on the right

**Figure 3:**
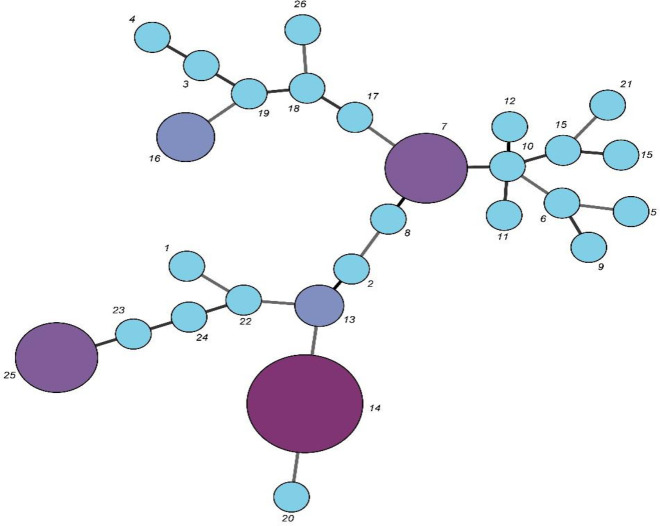
Minimum spanning tree of the 50 Multiple locus variable number of tandem repeats analysis (MLVA) typed *Bacteroides fragilis* isolates. MLVA profiles were clustered using categorical coefficient. In this tree, the MLVA types are illustrated as different colored circles. The size of each circle is indicative of the number of isolates with specific MLVA type

All isolates presented a PCR product for all VNTR loci. Diversity in the number of repeats between various VNTR loci was observed based on the analysis of all 8 VNTR loci of *B. fragilis*. Overall, *B. fragilis* isolates were distinguished into 27 different MLVA types (MT) as indicated in Figure 2. Types 1, 2, 3, 4, 5, 6, 8, 9, 10, 11, 12, 15, 16, 18, 19, 20, 21, 22, 23, 24, 25, and 27 only included one strain each. Type 13 included two strains. Type 17 contained three strains. Type 14 had 12 strains, making it the most prevalent type. Three main clusters called A, B, and C were observed by the dendrogram. Cluster A is categorized into two sub-clusters A1 and A2, sub-cluster A1 with four types and sub-cluster A2 with eight types. In addition, cluster B is divided into two sub-clusters B1 and B2, sub-cluster B1 with two types, and sub-cluster B2 with two types. Cluster C is divided into two sub-clusters C1 and C2, sub-cluster C1 with four types, and sub-cluster C2 with seven types. This study aimed to determine MLVA profiles of 50 strains from eight different origins to determine the relatedness of these isolates. These strains were distinguished into 27 types by the MLVA technique. All strains were isolated from the stools of healthy individuals. According to the cluster analysis of the MLVA profiles by a minimum spanning tree algorithm, MLVA was efficient in discriminating *B. fragilis* isolates from different sources as shown in Figure 3. The Simpson and Hunter index analysis showed the highest diversity in tandem repeats 5, 1, 8, 2, 6, 4, 3, and 7, respectively (Table 3). 

**Table 1:** Characteristics of variable-number tandem repeat loci used for typing of *Bacteroides fragilis* YCH46 isolate

**TRs**	**Repeat motif**	**TR Size **	**Genomiclocation**	**Function** ^a^	**Unit**	**PI ** ^b^
TR1	AACATCCGGATGTTTTAATATAT	23(bp)	349742--349830	non-coding region	4	0
TR2	GAAAACCATCAAGAAAGACATCTTTGGAGATACTGTCATTGAGGACAATCGCGGTAATAG	60 (bp)	1664955--1665296	hypothetical protein	6	0
TR3	ACGATAAGCGGTGA	14 (bp)	1762135--1762195	non-coding region	4	0
TR4	GAAGTGTAAAAGTAACAATCCGTCAAAGGAGACAGTGAAGGAAAGCCATAAGGCATATACGGTTC ACTGCCCATTCTCCATTTGCA	86 (bp)	3424647--3425269	hypothetical protein	7	1
TR5	AGCCGAAGTTACGGTGCTGCGT	22 (bp)	3745326--3745553	non-coding region	10	0
TR6	ATGACACAGTAA	12 (bp)	3978553--3978616	hypothetical protein	5	0
TR7	TCCTGACCGTCTTTACCATCGGTACCG	27 (bp)	4278063--4278232	hypothetical protein	6	0
TR8	CGGACGATAGGGACGGTCGCCACCTTCACGATTATATGAAGGACGTTGCGGACGGTCGCCATAAG AACGCTGAGGACGATCACCACCTTCGCTGTTAAAACG	102 (bp)	4586247--4586699	ribosomal large subunit pseudouridine synthase B	4	0

^a^ The role of TR-harboring gene, ^b^ Percent Indels

TR: Tandem repeats

**Table 2:** Primers and their characteristics in the current study

**TRs**	**Primer sequences 5’** **3’**	**PP ** ^a^ ** (bp)**	**Flanking (bp)**	**Reference**
TR1	F= TGAATACATTTCCTTTTTGCCTCT R=CCTACACCTTCCTTGTATATCTCCAT	208	119	in this study
TR2	F= CCCTCGGATAACAGGGAGTT R= TGCTTTTTCCCATGATTATCTTC	498	156	in this study
TR3	F= CTGTTCATTTTCGGACAGCA R= GCGGCTACTGATCTTTTAGCA	161	141	in this study
TR4	F= TCCGTCCTGATACGGATTCT R= AATCTGCCCTTTCCATACCC	694	71	in this study
TR5	F= AGCACGTAACCGAAATCACA R= ACGTCCGGAAAAGGAGATG	285	60	in this study
TR6	F= ACAGCAGTGTTCAAACGTCAA R= GGTTGCCAGCAGATTGAGA	150	86	in this study
TR7	F= GTCCGTACCGTCTGTTCCA R= ATGGCGAGAAAGGAGACGAT	245	56	in this study
TR8	F= TACGACGTATCGGACGTGAG R= GGCCGTGATGGTAACAGAAG	550	97	in this study

^a^ PCR product length in *Bacteroides fragilis* isolate

TR: Tandem repeats

**Table 3:** Simpson’s Diversity and Hunter-Gaston Diversity

**Locus**	**Diversity index**	**Confidence interval**	**K**	**Max (pi)**
TR:5	0.737	0.723 - 0.752	4	0.333
TR:1	0.661	0.651 - 0.672	3	0.373
TR:8	0.654	0.638 - 0.671	3	0.412
TR:2	0.650	0.610 - 0.690	4	0.490
TR:6	0.617	0.587 - 0.648	3	0.490
TR:4	0.498	0.491 - 0.506	2	0.529
TR:3	0.498	0.491 - 0.506	2	0.529
TR:7	0.457	0.420 - 0.494	2	0.647
TR:5	0.752	0.738 - 0.767	4	0.333
TR:1	0.675	0.664 - 0.685	3	0.373
TR:8	0.667	0.651 - 0.684	3	0.412
TR:2	0.663	0.623 - 0.703	4	0.490
TR:6	0.630	0.599 - 0.661	3	0.490
TR:4	0.508	0.501 - 0.516	2	0.529
TR:3	0.508	0.501 - 0.516	2	0.529
TR:7	0.466	0.429 - 0.503	2	0.647

Diversity Index (for VNTR data)=indicates any variation in the number of repeats at every locus. Within the of 0.0 (indicative of no diversity) to 1.0 (indicative of complete diversity)

Confidence Interval=The level of precision in the diversity Index, indicated as 95% upper & lower cases

K=Number of different repeats at this locus in sample set.

max(pi)=Fraction of samples with the highest repeat numbers in this locus (within the range of 0.0 to 1.0)

TR: Tandem repeats

## Discussion

The current study evaluated the genetic association between 50 *B.* *fragilis* isolates from the stools of different individuals in Tehran, Iran. For a better understanding of the features and varieties of the isolates, *B*. *fragilis* isolates were analyzed by MLVA typing, which is considered a valuable method for determining the diversity of bacterial populations in clinical isolates. MLVA is an inexpensive, uncomplicated, and efficient method whose results can be attained within a short period, usually faster than MLST and PFGE techniques (25).

Furthermore, over the past few years, typing has been inclined towards molecular techniques resulting in the development of new techniques such as MLVA. Thus, this new technique can be a promising substitute for previous techniques, including ribotyping, serotyping, and RFLP. *B.* *fragilis* is an anaerobic pathogen commonly isolated from clinical specimens with different virulence factors. Among the diseases caused by Bacteroides are cerebrospinal angiomas, meningitis, septic arthritis, inflammatory bowel, intestinal diseases, and soft tissue infections (26, 27).

Owing to the evolution and adaptation of *B. fragilis*, like other human bacterial flora, quantitative diversity for this bacterium was not observed in this study. A significant relationship was found between MLVA types and strains isolated from stools of different individuals. The limited number of isolates makes it difficult to interpret epidemiological data. Further studies are required to assess the efficacy of MLVA assay in *B. fragilis* especially in the toxigenic strains.

## Conclusion

This technique can provide quick and valuable information for researchers to evaluate pathogenicity, evolution, and epidemiological studies of this microorganism.
